# Nanoformulation innovations: Revolutionizing precision in migraine therapy

**DOI:** 10.22038/ijbms.2024.79824.17290

**Published:** 2025

**Authors:** Mohammad Ghasemi Narimani, Fatemeh Kalalinia, Somayeh Marouzi, Sara Gheshlaghi, Zahra Salmasi, Maryam Hashemi

**Affiliations:** 1 School of Pharmacy, Mashhad University of Medical Sciences, Mashhad, Iran; 2 Student Research Committee, Mashhad University of Medical Sciences, Mashhad, Iran; 3 Biotechnology Research Center, Pharmaceutical Technology Institute, Mashhad University of Medical Sciences, Mashhad, Iran; 4 Departments of Pharmaceutical Biotechnology, School of Pharmacy, Mashhad University of Medical Sciences, Mashhad, Iran; 5 Nanotechnology Research Center, Pharmaceutical Technology Institute, Mashhad University of Medical Sciences, Mashhad, Iran; 6 Departments of Pharmaceutical Nanotechnology, School of Pharmacy, Mashhad University of Medical Sciences, Mashhad, Iran

**Keywords:** Drug delivery, Migraine, Nanocapsules, Nanoformulation, Nanoparticles, Nanotechnology

## Abstract

**Objective(s):**

Migraine, a serious neurological disease that affects millions of people worldwide, is one of the most considerable burdens on the healthcare system and has significant economic implications. Even though various treatment methods are available, including medication, lifestyle changes, and behavioral therapy, many migraine sufferers do not receive adequate relief or experience intolerable side effects. Hence, the present review aims to evaluate the nanoformulation regarding migraine therapy.

**Materials and Methods:**

Between 2005 and 2024, specific keywords were used to search several databases, such as Pubmed, Google Scholar, and Scopus.

**Results:**

The nanoformulation field is an increasing field within nanotechnology that offers new solutions for treating migraine, including improving drug delivery, increasing therapeutic efficacy, and minimizing side effects. By combining nanoscale materials with therapeutic agents, nanoformulations can enhance bioavailability, sustain drug release, deliver targeted drugs, and penetrate the Blood-Brain Barrier (BBB) more efficiently. Nanoformulation has the potential to be a useful tool for migraine therapy. However, several challenges still need to be overcome, such as the BBB penetration, safety and biocompatibility of the product, manufacturing, and scalability reproducibility to pass regulatory approval and affordability. To overcome these challenges, research efforts should be focused on developing innovative techniques to penetrate the BBB, target specific migraine pathways, incorporate personalized medicine approaches, and develop nanotechnology-based diagnostics.

**Conclusion:**

A nanotechnology-based approach aims to revolutionize migraine therapy, improving patient outcomes and living standards by offering personalized and precise treatments.

## Introduction

Migraine is a serious neurological disease that causes weakening episodes of severe vomiting and headaches, accompanied by nausea and a sensitivity to light and sounds (1). There are approximately 1 billion people around the world affected by migraine, making it one of the most widespread and disabling conditions in the world (2). Despite the significant impact migraine has on a person’s quality of life and productivity in the workplace, migraine remains an ununderstood and undertreated condition. There are a variety of neurobiological, genetic, and environmental factors that contribute to migraine’s pathophysiology (3). It is widely believed that abnormal neuronal excitability and a malfunction in pain processing pathways in the brain cause migraine. In migraine attacks, several neurotransmitters influence the development and proliferation, including dopamine, serotonin, and Calcitonin Gene-Related Peptide (CGRP) (4). The severity and frequency of migraine attacks have been reduced over time by a variety of treatment modalities.

There are several drugs available to treat migraine, such as triptans, Non-Steroidal Anti-Inflammatory Drugs (NSAIDs), ergot alkaloids, as well as preventive medication such as Antiepileptic Drugs (AEDs), beta-blockers, and antidepressants (5). Moreover, stress management techniques, complementary therapies like biofeedback, acupuncture, and lifestyle modifications have been advocated for the treatment of migraine (6). Even though there are many treatment options available to migraine sufferers, many of them still experience inadequate relief from their migraine or experience intolerable side effects as a result of conventional treatments (7). Long-term use of certain migraine medications can also cause rebound headaches, drug resistance, and other side effects, so alternative migraine management approaches are essential (8). Recently, interest has increased in developing novel drug delivery systems to improve migraine treatment safety, compliance, and efficacy (9).

Nanotechnology-based formulation has emerged as a promising candidate for targeted drug delivery to migraine patients’ Central Nervous Systems (CNS) (10). Using nanoformulations in drug delivery can revolutionize the treatment of migraine by leveraging the impressive properties of nanoscale materials (11). Nanotechnology involves manipulating particles smaller than 100 nanometers, a scale at which materials exhibit distinct interactions and behaviors with living organisms (12). Using nanocarriers as sophisticated vehicles to encapsulate therapeutic agents enables agents to be shielded from degradation, facilitates their targeted delivery to specific anatomical sites in the body, and ensures that they reach the desired locations (13). A significant advantage of nanoformulations is that they can overcome traditional drug delivery systems’ inherent limitations. As a result of oral and parenteral administration routes, drug bioavailability is often poor, clearance from the circulation is rapid, and off-target effects are frequent, resulting in frequent dose administrations to achieve therapeutic concentrations at the intended site of action (14). In contrast, nanocarriers can enhance drug stability, prolong drug release kinetics, and enhance tissue penetration, thus improving therapeutic efficacy while minimizing systemic toxicity (15). Moreover, nanoformulations can modulate drug biodistribution profiles and pharmacokinetics with unparalleled versatility (16).

Researchers can tailor the behavior of Nanoparticles (NPs) in biological environments by adjusting their physicochemical properties, such as their surface charge, size, surface chemistry, and shape (17, 18). By controlling physicochemical properties, the design of nanocarriers can optimize pharmacokinetic profiles, leading to sustained drug release over long periods, less frequent dosing, and improved therapy compliance. (19). Migraine therapy relies heavily on nanocarriers’ ability to cross physiological barriers, such as the Blood-Brain Barrier (BBB) (20). Many migraine medications are ineffective due to the BBB, which prevents therapeutics from reaching the CNS. Nevertheless, nanocarriers have the potential to bypass the BBB altogether or exploit endogenous transport mechanisms, thus providing targeted delivery of drugs to the brain parenchyma (21). The targeted delivery method promotes drug accumulation at the pathological site while minimizing systemic exposure, reducing systemic adverse effects (22). 

Researchers can harness nanotechnology’s power to develop precision, productive approaches to migraine management. The present review aims to overview the current migraine treatment landscape and the challenges and potential benefits of nanoformulation approaches. The discussion will focus on the efficacy and safety of nanoformulated migraine treatments and the various nanocarriers and delivery strategies used to deliver targeted drugs to the CNS. The final objective will be to improve the quality of life and patient outcomes by applying nanotechnology to migraine management. 

**Methodology**

Between 2005 and 2024, we conducted a comprehensive literature review using specific keywords, including “nanoformulation,” “precision therapy,” “migraine treatment,” “drug delivery,” and “nanomedicine.” The primary databases consulted were PubMed, Google Scholar, and Scopus. These sources were chosen for their extensive and reputable scientific content. Relevant studies, reviews, and clinical trials were meticulously selected and analyzed to gather data on the advancements and applications of nanoformulations in precision migraine therapy. The collected information was synthesized to provide a comprehensive overview of current innovations and prospects in the field.

**Overview of migraine**

Migraine is a multifaceted neurological disease. There are approximately 1 billion people who suffer from the disease worldwide, making it a prevalent and disabling condition. Despite migraine’s widespread impact, researchers and clinicians still struggle to understand its pathophysiology, etiology, and best management strategies (23). Symptoms of migraine include severe headaches accompanied by nausea, vomiting, photophobia, and phonophobia (24). The migraine manifests in different ways and exhibits considerable variability in symptoms, duration, and severity over time. Migraine are episodic headaches characterized by recurrent headache episodes separated by intervals of symptom-free time between attacks (25). It is common for migraine attacks to range in severity and frequency from occasional mild headaches to frequent, debilitating attacks that significantly compromise daily life (26).

There are several factors associated with migraine pathogenesis, including neurobiological, genetic, and environmental factors that contribute to the development of migraine symptoms (27). Researchers have found that migraine is associated with abnormalities of the peripheral and CNS as well as altered neurovascular function, cortical excitability, and neurotransmitter signaling (28). Migraine attacks are initiated, propagated, and modulated by these aberrations, which ultimately result in the patient’s characteristic symptoms (29). The pathophysiology of migraine often involves Cortical Spreading Depression (CSD), characterized by a transient period of neuronal hyperactivity followed by a period of neuronal depression (30). There are a variety of subtypes, such as chronic migraines, migraine with aura, and migraine without aura. The International Classification of Headache Disorders (ICHD) covers migraine subtypes. Various migraine attack characteristics, including preceding aura symptoms, duration, intensity, and frequency, are used to diagnose migraine attacks (31). A migraine treatment strategy has the purpose of improving acute migraine symptoms, preventing migraine attacks, and improving the overall quality of life of patients with migraine. There are several acute treatment options available, including analgesics such as ergot alkaloids, NSAIDs, and triptans, which target the pain pathways behind migraine headaches as well as the vascular mechanisms that contribute to them (32).

Migraineurs experiencing the aura phase, which includes reversible neurological symptoms like sensory disturbances, visual disturbances, and motor deficits, are believed to harbor CSD. Although the exact mechanisms triggering headache pain associated with CSD remain unclear, they may involve activating trigeminal nociceptive pathways and releasing pro-inflammatory mediators (33). Additionally, the pathogenesis of migraine has been thought to be related to abnormal regulation of neurotransmitter systems, such as dopamine, CGRP, and serotonin (34).

A disruption of serotonin receptor function is thought to contribute to migraine susceptibility, with serotonin being a critical factor in modulating neuroinflammation, pain perception, and vascular tone (35). Additionally, dysfunctions in the CGRP and dopaminergic signaling pathways have been linked to progression and migraine onset, highlighting the complex interactions between neurotransmitter systems (36). Migraine is also strongly affected by genetic predisposition, with heritability estimates and familial clustering suggesting a vital genetic component. Many genetic variants have been identified as being linked to migraine risk by Genome-Wide Association Studies (GWAS), most of which are associated with synaptic transmission, neuronal excitability, and ion channel function. However, migraine is a highly complex disease with a highly complex genetic architecture (37). Multiple genetic loci are implicated in the disease’s susceptibility and the phenotype’s variability. Besides genetic factors, environmental triggers and lifestyle factors may also play a part in precipitating migraine attacks or causing them to become worse (38).

The most common triggers are weather changes, hormonal fluctuations, stress, alcohol consumption, sleep disturbances, sensory stimuli (such as loud noises, strong odors, and bright lights), environmental toxins, and caffeine consumption (39). Although there is no clear understanding of the mechanisms involved in triggering migraine attacks, it is believed that triggers modify vascular tone, neuronal excitability, neuroinflammatory responses, and ultimately resulting in triggering or exacerbating migraine-related symptoms. Due to the lack of specific biomarkers and diagnostic tests that can be used to diagnose migraine, clinical diagnosis still needs to be improved due to the reliance on patient reports of their medical history and symptoms (40). 

As another treatment option, migraine patients may be prescribed antiemetic medications to reduce vomiting and nausea caused by migraine attacks. As a result, acute migraine treatments often lack efficacy and tolerability, as well as their potential to cause Medication Overuse Headaches (MOH), which calls for alternative approaches to migraine management (41). Migraine treatment has recently undergone a revolution with the advent of targeted migraine therapies, such as monoclonal antibodies that target CGRP or its receptors, which provide more effective and specific treatments for both preventive and acute migraine (42). As compared with conventional pharmacological treatments, biologic agents have the potential to reduce migraine attacks’ severity and frequency, improving outcomes and minimizing adverse effects (43). Furthermore, non-pharmacological treatments for migraine, such as lifestyle modification, behavioral therapy, and neuromodulation, provide additional options for personalized treatment (44). Patients who undergo migraine can find treatment modalities available, but many still experience poor relief, refractoriness to treatment, or intolerable side effects, which calls for innovative approaches to treat migraine (45). As nanotechnology-based drug delivery systems become more prevalent in recent years, nanoformulations show great potential for improving migraine therapies’ efficacy, safety, and adherence. In the future, nanoformulations can overcome the limitations of conventional drug delivery systems by encapsulating therapeutic agents in nanocarriers and optimizing their physicochemical properties. Nanoformulations can improve migraine medications’ targeted delivery to the CNS, enabling them to exert their therapeutic effects more precisely and efficiently (46, 47). 

**Current treatment options for migraine and their limitations**

**Figure 1:**
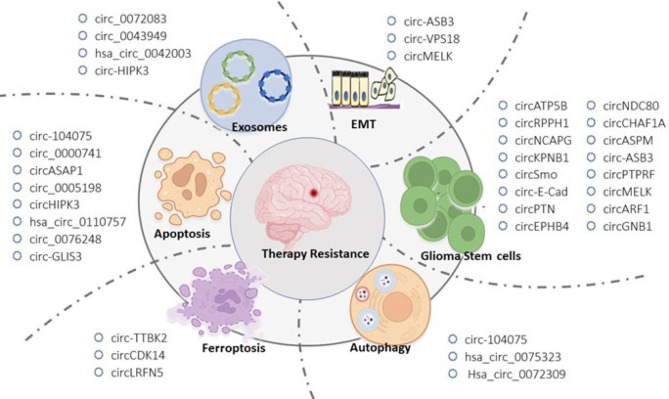
Current treatment options for migraine

Despite being a weakening neurological disease, migraine presents significant challenges for healthcare providers and patients because of their complex heterogeneous and pathophysiology clinical presentations. Various treatments can reduce migraine symptoms and minimize their severity and frequency (Figure 1), but they are often limited in their adverse effects, efficacy, and tolerability. 

***Acute treatment options***

*Nonsteroidal anti-inflammatory drugs*

Aspirin, naproxen, and ibuprofen are commonly used as NSAIDs to treat acute migraine pain by reducing inflammation and suppressing prostaglandin synthesis (48). Some migraine sufferers have found relief through the use of NSAIDs. However, their efficacy may be limited when the migraine is severe or refractory, and prolonged treatment may result in gastrointestinal adverse effects such as bleeding and ulcers (49). 

*Triptans*

In particular, triptans are a group of medications that target migraine symptoms by attaching themselves to the receptors of serotonin in the brain and constricting the blood vessels that are dilated (50). Migraine attacks can be effectively prevented, and symptoms such as photophobia and nausea are alleviated with triptans (51). However, the use of triptans is limited by the possibility of cardiovascular problems such as stroke, coronary artery spasm, and myocardial infarction, particularly in those who have preexisting cardiovascular issues. 

*Ergot alkaloids*

Acute migraine attacks have been treated for decades with ergot derivatives such as Dihydroergotamine (DHE) and ergotamine (52). Several medications can increase serotonin levels and inhibit the release of vasoactive peptides, but they exert their effects differently (53). Ergot alkaloids have been proven to be effective in averting migraine attacks, but they can cause significant side effects, including nausea, vasoconstriction of peripheral vessels, and vomiting. 

*Analgesics*

In mild to moderate migraine pain, Over-The-Counter (OTC) analgesics such as acetaminophen and combinations of acetaminophen and caffeine or codeine may relieve symptoms (54). The efficacy of OTC analgesics in treating migraine attacks that are severe or persistent is limited, and long-term use may become associated with rebound headaches and MOH (55).

*Calcitonin gene-related peptide-targeting drugs for migraine*

Calcitonin gene-related peptide (CGRP) has emerged as a pivotal target in migraine therapeutics, leading to the development of CGRP inhibitors as promising treatments (56). Understanding the pharmacology of these drugs is crucial for informed treatment decisions. CGRP inhibitors, including monoclonal antibodies and small molecule antagonists, act by blocking the CGRP pathway, thereby reducing the frequency and severity of migraine attacks (57). Their efficacy in clinical trials underscores their potential as a novel therapeutic approach for migraine management (58). However, despite their promise, CGRP inhibitors may pose potential off-target effects, including cardiovascular risks and liver toxicity, necessitating careful patient selection and monitoring (59). Moreover, ongoing research is shedding light on the broader implications of CGRP in migraine pathophysiology, suggesting potential future directions for therapy (60). While CGRP inhibitors represent a significant advancement in migraine treatment, clinicians must weigh their benefits against potential risks and consider individual patient factors to optimize therapeutic outcomes and ensure patient safety (61, 62).

***Preventive treatment options***

*Beta-blockers*

A beta-blocker, such as timolol, propranolol, and metoprolol, is commonly prescribed as a first-line migraine preventive medication because it reduces neuronal excitability and inhibits sympathetic nervous system activity (63). In addition to reducing migraine severity and frequency, beta-blockers may also cause depression, cardiovascular adverse effects, and fatigue, which may limit their use (64).

*Antiepileptic drugs*

Valproate, gabapentin, and topiramate are AEDs that modulate neurotransmitter release, inhibit cortical hyperexcitability, and stabilize neuronal membranes to prevent migraine attacks (56, 57). However, adverse effects associated with AEDs can impact the central nervous system (CNS), leading to mood disturbances, cognitive impairment, and dizziness, which can limit long-term adherence and tolerability (65).

*Calcium channel blockers*

Several calcium channel blockers, such as flunarizine and verapamil, have been investigated to determine if they may prevent migraine headaches by modulating the release of neurotransmitters and preventing calcium from entering neurons (66, 67). In addition to reducing migraine severity and frequency, calcium channel blockers may cause adverse cardiovascular effects such as peripheral edema, hypotension, and bradycardia (68, 69).

*Botulinum toxin injections*

It has been suggested that Botulinum toxin type A (Botox) injections can be used as a treatment option for chronic migraine, which is defined as migraine occurring 15 days or more a month (70). The effects of Botox are attributed to the inhibition of the release of neurotransmitters involved in the transmission of pain, as well as the reduction of peripheral sensitization and muscle hyperactivity (71). A Botox injection can significantly reduce migraine symptoms for some chronic migraine. However, their effectiveness varies from person to person and must be repeated every 12 weeks (72). 

***New drugs***

The newest drugs for the acute treatment of migraine include Zavzpret (zavegepant) nasal spray, approved in March 2023, and Nurtec ODT (rimegepant) and Ubrelvy (ubrogepant), orally-administered calcitonin gene-related peptide (CGRP) receptor antagonists (gepants), approved in 2020 (73, 74). Additionally, Reyvow (lasmiditan), the first serotonin (5-HT) 1F receptor agonist, offers a novel mechanism for migraine relief. New formulations of older drugs like sumatriptan and rizatriptan, which are serotonin (5-HT) receptor agonists (triptans), have also been approved. For migraine prevention, recent advancements include Qulipta (atogepant), an oral CGRP antagonist approved in September 2021 for episodic migraine, and monoclonal antibody CGRP antagonists such as Aimovig, Ajovy, Emgality, and Vyepti (75-78).

**Introduction to nanoformulation**

There are various migraine treatment options available, but most migraine patients do not experience adequate relief, treatment refractoriness, or intolerable side effects from the current therapies. Moreover, migraine sufferers frequently have comorbidities, overuse of medications, and varying responses to treatment. As a result, it is essential to develop new therapeutic strategies targeting migraine’s underlying pathophysiology while optimizing patient outcomes and minimizing adverse effects (79). Using nanoformulations to overcome these challenges will be discussed in the next section. The development of nanotechnology as a method for drug delivery has emerged as a promising frontier for various medical conditions, including migraine, that currently lack effective treatment modalities (80). In nanotechnology, nanoformulation involves the development of nanoscale drug delivery systems designed to encapsulate, protect, and deliver therapeutic agents to specific body locations (81). Using the unique physical and chemical characteristics of NPs, researchers are developing migraine medications that will improve therapeutic efficacy, pharmacokinetics, and biodistribution (82). Nanoformulation aims to manipulate particles with sizes ranging from 1 to 100 nanometers, a scale where materials behave differently and interact differently with the body (83, 84). Many materials can be used as nanocarriers, including metals, lipids, inorganic and polymers NPs, each with its benefits regarding stability, drug density, and biocompatibility (85-87). Using nanocarriers as sophisticated delivery vehicles, therapeutic agents can be encapsulated, protected from degradation, and delivered efficiently to cell types or specific tissues (88). 

***Nanoformulations advantage*** **s**

Nanoparticles can improve bioavailability by enhancing poorly permeable or soluble drugs’ absorption, dissolution rate, and solubility (89). It has been shown that hydrophobic drugs may be encapsulated within lipid-based NPs or conjugated to hydrophilic polymers to overcome the challenges associated with the delivery of hydrophobic drugs and maximize therapeutic effects (90, 91).

*Sustained release kinetics*

With nanoformulations, drugs can be designed to have sustained release kinetics, prolonging their action and reducing dosing frequency (92). Researchers can modulate the surface properties, size, and shape of NPs to control the rate at which drugs are released and optimize the profile of their pharmacokinetics to achieve extended therapeutic concentrations (93, 94). 

*Targeted drug delivery*

The surface of target cells can be addressed with particular targeting ligands, including aptamers, antibodies, and peptides, or to facilitate specific binding (95, 96). This method can precisely deliver therapeutic agents to diseased tissues or organs with a minimum effect on healthy tissues, improving therapeutic efficacy and minimizing systemic adverse effects (97, 98). 

*Crossing biological barriers*

In addition to the BBB, nanoformulations can penetrate the mucosal epithelium, enabling therapeutics to be delivered to previously inaccessible anatomical sites (99). Pharmacologists can enhance drug penetration into target tissues by engineering NPs with specific physicochemical properties or exploiting endogenous transport mechanisms (100, 101). 

**Nanoformulation approaches for migraine treatment**

**Figure 2:**
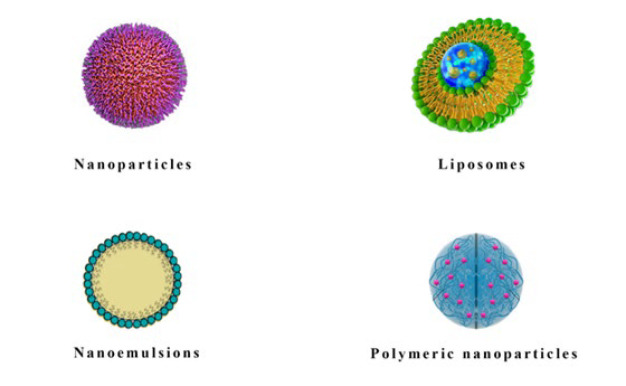
Nanoformulation approaches for migraine treatment

The use of nanoformulations in migraine therapy offers a promising means of improving the efficacy and delivery of existing migraine medications while minimizing side effects. Several nano formulation methods have been investigated for migraine treatment (Figure 2), which will be discussed below. 

***Lipid-based nanocarriers***

The potential application of lipid-based NPs in migraine therapy has gained significant attention, including Nanostructured Lipid Carriers (NLCs), liposomes, and Solid Lipid Nanoparticles (SLNs) (102). Nanocarriers can encapsulate a wide range of hydrophilic and hydrophobic drugs, such as trigeminal ganglion antagonists, NSAIDs, and triptans, and deliver them to the brain parenchyma, where they will exert their therapeutic effects (103). As a drug delivery vehicle for migraine treatment, lipid-based nanocarriers have several advantages, including stability, biocompatibility, and combining hydrophilic and lipophilic drugs (104). 

***Polymeric nanoparticles***

The use of polymeric NPs for migraine therapy has been demonstrated as a versatile platform for delivering controlled drug delivery using biodegradable polymers, such as chitosan, Poly Ethylene Glycol (PEG), and Poly Lactic-co-Glycolic Acid (PLGA) (105). A wide range of therapeutic agents can be encapsulated with NPs, protected from degradation, and released over a prolonged period (106). Using polymeric NPs, migraine medications can be designed to target specific cells or tissues in the CNS, such as astrocytes or microglia, that play an important role in migraine pathogenesis (107). 

***Nanoemulsions***

There are certain types of nanoemulsions, for example, the nanoemulsions of oil and water, which surfactants have stabilized to encapsulate hydrophilic or lipophilic drugs inside nanoscale droplets. With nanoemulsions, migraine therapy can be improved through enhanced gastrointestinal absorption, increased drug solubility, and systemic delivery of drugs to the central nervous system (108, 109). Depending on patient preference or clinical consideration, nanoemulsions can be administered orally, intravenously, or intranasally, providing flexibility and tailored treatment approaches (110-112).

**Recent studies**

***Curcumin***

A study in 2021 showed there was a significant reduction in migraine attack frequency and serum levels of Interleukin 1 Beta ( IL-1β) between individual treatments and the combination of n-3 fatty acids and nano-curcumin (*P*<0.001) (113). However, after numerous experimental modifications, its importance was lost, and there was a significant reduction in IL-1β gene expression between the combination and individual groups (*P*<0.05). 

A 2019 study conducted to evaluate the combination of liposomal curcumin and naproxen improved anti-oxidant and anti-nociceptive actions compared to naproxen alone or curcumin solution in rat migraine models, suggesting a promising therapeutic approach (114). Notably, there was a significant reduction in oxidative stress markers and pain between a combination of naproxen (2.8 mg/kg) with liposomal curcumin (2 mg/0.1 kg) and naproxen groups (*P*<0.001). 

Abdolahi *et al*. (2019) conducted a study to examine the association between migraine and recurrent pain attacks with neuroinflammatory processes involving the Cyclooxygenase (COX-2) and Inducible Nitric Oxide Synthase (iNOS) pathways (115). The omega-3 fatty acids and curcumin contain anti-inflammatory properties that target the expression of iNOS/COX-2. An analysis of 74 migraine patients was conducted in the study. There was a significant reduction in iNOS/COX-2 levels and clinical symptoms between omega-3 and nano-curcumin (*P*<0.05). As a result, the treatment was found to be promising for the prevention of migraine. Table 1 summarizes the studies about curcumin nanoformulations used for migraine. 

***Zolmitriptan***

Zolmitriptan-loaded PLGA NPs have shown a 14.13-fold increase in brain delivery compared to the free drug in animal studies (46). These NPs also significantly reduced hyperalgesia and photophobia in migraine models (Swiss albino mice).

Moreover, a 2017 study showed the low bioavailability of Zolmitriptan (ZMT), which effectively treats migraine (112). Nasal mucoadhesive nanoemulsion formulations have been developed to enhance direct delivery to the brain for high concentrations in the brain and rapid action. An investigation of the viability of ZMT mucoadhesive nanoemulsions was conducted, which included evaluating and preparing drug content, morphology, zeta potential, residence time, particle size, and viability of nasal mucosa permeation. An in vivo study in mice compared its pharmacokinetics in mice with intravenous and nasal solutions, finding that the mucoadhesive nanoemulsion produced an improved brain Area Under the Curve (AUC 0-8) and a lower Time to peak drug concentration (Tmax) due to its more excellent permeability and small globule size. 

Jha *et al*. (2022) conducted a study to examine mucoadhesive polymeric NPs of ZMT for intranasal administration to improve brain targeting and bioavailability during migraine attacks (116). With ZMT NPs, there is a significant increase in absolute bioavailability (193%) and better nasal-to-brain transport than with oral drug administration. It was found that adult male Swiss albino mice could tolerate reduced abdominal stretching and bright light in response to the treatment, indicating that the treatment is effective in reducing migraine symptoms. Intranasal delivery of NPs of ZMT appears to be a promising migraine treatment approach. 

A 2020 study examined the Wistar albino rats given nasal sprays of chitosan NPs containing ZMT for pharmacokinetic studies (117). There was a significant increase in drug levels in plasma and brain tissue in the test formulation compared to the group receiving water for injection or standard drug solutions. Specifically, the test formulation shows a significant increase in the plasma concentrations (41.37 ± 2.31 ng/ml) compared to standard groups at 10 min post-administration (*P*<0.05). However, there was a significant increase in levels of drug absorption in brain tissue (15 ± 0.08 ng/g) compared to standard groups at 60 min post-administration (*P*<0.05). The findings indicate that nasal spray containing chitosan NPs loaded with ZMT may be effective at treating migraine headaches rapidly. 

Researchers in 2022 performed a study to investigate SLNs to enhance ZMT delivery across the BBB (118). It has been found that SLNs possess favorable characteristics, such as high drug entrapment (84.17 x 12.24%), small size (110-200 nm), and stability, providing significant drug release both in vitro (95.85 x 2.44%) and in vivo (82.06 x 2.94%) over 24 hr. In vivo studies in male Wistar rats demonstrated enhanced pharmacokinetic parameters AUC (37.05 ± 2.45 ng/ml), Cmax (42.08 ± 1.32 ng/ml), and Tmax (30 min), t1/2 (1.28 hr)). Pharmacokinetic parameters indicate the effectiveness of intranasal delivery of ZT-loaded SLNs to bypass the hepatic metabolism, which improves the bioavailability and permeation of the drug, compared to oral administration. 

Girotra *et al*. (2016) showed a quality-based design approach; PLGA/poloxamer NPs of ZMT were developed for brain delivery (106). In the optimized NPs, the size ranged from 165.4–245.4 nm, the encapsulation efficiency was 48.96-94.97%, and the drug release ranged from 43.32 to 100%. An analysis of the drug loading confirmed that there were no interactions between the drugs. The drug loading was confirmed to be successful without interactions based on the characterization. In vivo studies demonstrated a 14.13-fold increase in brain uptake compared to free drug, leading to significantly enhanced anti-migraine potential in Swiss albino mice. The findings suggest that ZMT-loaded PLGA/poloxamer NPs could effectively treat migraine. 

A study conducted in 2018 examined and characterized nanostructured polymeric carriers for the direct administration of ZMT to the nose as a nose-to-brain target for migraine treatment (119). Compared to intravenous and intranasal ZMT solutions, radiolabeled nanocarriers demonstrated improved characteristics and better brain uptake when administered via intranasal routes. The bimodal SPECT-CT scintigram results indicated that intranasal ZMT-loaded nanocarriers showed significant brain accumulation, suggesting their potential as migraine treatment systems. 

Mohamed *et al*., 2019 performed a study investigating a liposomal formulation of ZMT for enhanced transdermal delivery, addressing systemic conditions like migraine and topical infections like mycotic (120). In addition to demonstrating small vesicle sizes (133.1 nm) and high entrapment efficiency (88.7%), the optimized formulation (F11) showed improved drug release. The F11-loaded emulsion gel significantly improved pharmacokinetic parameters and drug permeation over plain ZMT-loaded emulgel, suggesting it could treat various diseases.

A 2010 study examined micellar nanocarriers developed for nasal delivery of ZMT, and the transport pathway of the drug was investigated (121). A micellar nanocarrier was designed to deliver ZMT through the nasal passages, and its transport pathway was explored. A toxicity study in rats demonstrated no risks associated with nasal administration. Research on biodistribution demonstrated superior brain targeting over intravenous and nasal administration. A brain localization and autoradiography study has suggested that the drug labeled for consumption travels from the nose to the brain. The developed nanocarrier appears to be a promising vehicle for targeting ZMT in the brain. 

Researchers in 2020 conducted a study to investigate a polymeric form of ZMT/chitosan nanostructured liposomes (ZT/CT NLCs) that are coated with Tween 80 to address the challenges associated with the use of ZMT (122). This formulation displayed high performance in terms of entrapment efficiency (78.14%), yield (60.19%), stability (0.28 mV), and negative zeta potential (-25.5 mV), resulting in good entrapment efficiency and yield. An application of NLCs to hard gelatin capsules in situ gelled for 30 hr and displayed pharmacological effectiveness for eight hours in mice, suggesting potential for improving other class III drugs’ efficacy.

A 2022 study intended to increase the bioavailability of ZMT. This medication falls into class III of the BCS, as NLCs are used as a transdermal delivery system (123). Based on a factorial design, NLC9 was optimized to be the most favorable particle size, to exhibit the highest entrapment efficiency, and to release the most drug. By incorporating NLC9 into a gel, application stability and portability were enhanced. According to pharmacokinetic studies conducted in rabbits, ZMT has a 1.76-fold greater bioavailability than oral ZMT. A histopathological safety confirmation was obtained, suggesting that NLCs could benefit transdermal ZMT delivery and improve drug bioavailability.

A study (2021) showed the efficacy of ZMT while minimizing adverse effects by developing self-nano-emulsifying drug delivery systems for ZMT incorporating lavender oil (ZMT-SNEDDS) (124). As a result of the complete factorial design, the size of globules and zeta potential were optimized. ATR-FTIR monitoring of the optimized formulation (F5) confirmed superior dissolution and permeation, demonstrating safety in acute toxicity testing. Efficacy studies conducted on migraine rats have shown that F5 can significantly improve psychological state and pain relief while normalizing brain activity. 

In 2016, a study examined ZMT-loaded nanoliposomes added to a thermos-reversible gel and mucoadhesive polymers added to facilitate nose-to-brain drug delivery via mucoadhesive polymers (125). Ethosomes with excellent characteristics were obtained, including formulation E6, which has an entrapment efficiency (66%) and optimal size (171.67 nm). A thermos-reversible gel based on poloxamer 407 showed sufficient phase transition temperatures. According to the histopathological analysis of the optimal formulations G3 and G6, these formulations demonstrated promising gel characteristics, *in vitro* release, *ex vivo *permeation, and non-toxic effects. Table 2 provides a summary of each study included in the review.

***Sumatriptan***

Hansraj *et al.* performed a study investigating sumatriptan succinate-containing SLNs developed for targeting the brain using chitosan SLNs (126). A complete factorial design was utilized to optimize the formulations so that all particle sizes and zeta potentials are minimized, entrapment efficiency is maximized, and the brain-to-plasma ratio is maximized. In rats, optimized SLNs increased the brain/blood drug ratio by 4.54-fold, indicating successful brain targeting. Various analytical techniques were used to characterize the formulation and confirm its efficacy and integrity. Ultimately, the study suggests that delivering sumatriptan succinate orally is an effective method of managing migraine. 

Patravale *et al*., 2019 studied the advent of nanoformulations, which have attracted tremendous attention in recent decades due to their application in improving drug bioavailability (127). Microgels have several advantages, such as thermodynamic stability, ease of formulating, and enhanced penetration of biological barriers. These advantages make micellar nanosystems extremely useful in oral, transdermal, and parenteral administration. Additionally, they are being explored as noninvasive means of delivery, such as nose-to-brain. The purpose of this chapter is to describe a protocol for preparing sumatriptan-loaded micelles to treat migraine headaches. Micelles contain hydrophobic regions of diblock polymer that hold the drug and hydrophilic regions that provide conformational stability in aqueous environments.

Masjedi *et al.* performed a study to investigate a solution to the limitations of oral administration for migraine; sumatriptan-loaded NLCs were developed to deliver the drug nose-to-brain (128). Based on optimization, the NLCs had a mean diameter of 101 nanometers and a drug entrapment efficiency of 91.00%. Following intranasal administration, in vivo pharmacokinetic evaluations in rats showed significant Drug Targeting Efficiencies (DTEs) and Direct Transport Percentage (DTPs), indicating that NLC formulations have potential for brain delivery. 

Researchers studied (2022) Nanoliposomes (NLs) coated with chitosan (CCLs), which were developed to enhance the bioavailability of the sumatriptan succinate (SS) in humans (129). The formulations were optimized by investigating their physicochemical properties and the associated pharmacokinetic parameters. Compared to SS and SS-NL, CCLs showed faster absorption and a shorter tmax than SS and SS-NL. The chitosan-coated NLs enhanced the absorption of SS, suggesting that drugs are delivered to the systemic circulation more effectively. According to the animal model, liposomal and chitosan formulations demonstrated better kinetic behavior than soluble forms. 

Yadav *et al*. investigated Sumatriptan succinate SLNPs, which were developed as nasal delivery vehicles for the drug (130). A central composite design was used using solvent injection to optimize formulation to minimize particle size, optimize zeta potential, and ensure maximum entrapment. Optimized batch parameters included 133.4 nm particle size, -17.7 mV zeta potential, and 75.5% entrapment efficiency. In the laboratory, the particle morphology was spherical, and the release of the drug was sustained for 12 hr. Diffusion studies *in vivo* showed rapid permeation across the nasal mucosa, indicating a brain target. It was confirmed histopathologically that the nasal mucosa was intact after treatment. As a result, SLNPs may be a promising delivery method for sumatriptan succinate nasally. 

Girotra *et al*., in 2016, performed a study to optimize the performance of PBCA NPs for the delivery of sumatriptan succinate to the brain and compare them to SS-loaded BSA-ApoE NPs (131). A central composite design optimizes the size and release of the PBCA NPs. There was a higher brain uptake of SS-AA-NP in rats than in FPopt in *in vivo* studies. Mice behavioral tests confirmed SS-AA-NP’s superior antimigraine potential. There is a potential for better brain targeting of SS with BSA-ApoE NPs in migraine treatment. 

In 2018, a study was conducted to investigate the effects of liposomal curcumin at a dose of 2 mg/100 g body weight in combination with sumatriptan in an experimental migraine model induced by nitroglycerin in rats (132). Researchers demonstrated significant reductions in plasma total oxidative stress levels, malondialdehyde levels, and nitric oxide levels following the treatment. Moreover, liposomal curcumin was found to have superior antioxidative properties compared with curcumin solution, which suggests that liposomal curcumin could be an optimal treatment method for migraine, warranting further research.

A 2020 study examined an intranasal formulation containing copaiba oil and biopolymers developed to enhance the delivery of sumatriptan in treating migraine (133). A stable Nano Emulsion (NE) that demonstrates favorable properties after over a year of use can prolong sumatriptan release by more than 24 hr. An in vivo test in zebrafish confirmed the safety of both the alginate-based NE and its potential efficacy, suggesting that it may be able to treat migraine pain. 

In 2017, researchers investigated the hydrothermal synthesis of magnetic porous carriers based on tin oxide nanocrystals (134). This material had a high magnetization and a uniform particle size of 65 nm. Studies showed that the drug release of Sumatriptan, an anti-migraine drug, can be efficient and controlled remotely. The optimal drug efficiency (70%) was achieved with specific parameters. *In vitro *studies and stability testing confirmed that the carrier is appropriate for in vivo applications. Table 3 shows a summary of sumatriptan nanoformulations used for migraine.

**Others**

Harjot *et al*. performed a study to investigate an aerosol drug delivery system for flunarizine dihydrochloride that would improve solubility and therapeutic effect (109). A compatibility study confirmed the formulation’s suitability (110). Moreover, Nystatin-loaded chitosan NPs administered intraperitoneally in rats also demonstrated enhanced brain targeting and reduced migraine-associated behaviors like hyperalgesia, photophobia, and phonophobia compared to free drug (135). This represents a novel approach using the antifungal drug nystatin for migraine treatment.

Dali *et al*. conducted a study to examine ergotamine and caffeine hybrid NPs that were PEGylated lipid-polymer hybrids, focusing on controlled release, entrapment efficiency, and permeability (107). Nanoparticles produced using a single-step nanoprecipitation method had favorable characteristics, including good stability, small size (239.46 2.31 nm), controlled release profile, and high entrapment efficiency (86.88 1.67%). *In vitro* and *ex vivo* studies indicate sustained release over 48 hr following intranasal administration and significant brain uptake post-intranasal administration, with histopathologic and toxicological studies confirming safety and anti-hyperalgesia. There was potential for the formulation to be used to treat migraine.

Furthermore, sumatriptan succinate-loaded chitosan NPs developed using the Taguchi optimization method showed favorable characteristics for intranasal delivery, including a mean size of 306.8 nm, positive zeta potential of +28.79 mV, and 75.4% drug entrapment efficiency (136). The *in vitro* release of sumatriptan from these NPs through goat nasal mucosa was 76.7% over 28 hr, indicating their potential for improved bioavailability and therapeutic effect compared to the free drug.

Abou Youssef *et al*. performed a study to investigate a safe and efficient system for delivering the water-soluble anti-migraine medicine Almotriptan Malate (ALM) intranasally (137). As a result of a double-emulsion-solvent evaporation process, SLNs were prepared and optimized for entrapment efficiency, polydispersity index, and particle size. A mucoadhesive in situ gel contained Na-CMC and Poloxamer 407, which included the optimized SLNs dispersed within the gel. ALM was rapidly delivered into the brain through the formulated system, with promising results in both in vitro and in vivo studies and favorable toxicological risk profiles. As a result of these findings, it is possible to conduct future clinical trials using the delivery system developed in humans with positive results. 

A 2020 study investigated whether chitosan-coated NLCs can effectively deliver Almotriptan maleate (ALM) through nasal mucosa (138). During formulation optimization, the final formula (F1) showed favorable properties regarding zeta potential (34.1 mV), particle size (255 nm), entrapment efficiency (80%), and PDI (0.27). Studies on the compounds’ in vivo and in vitro release and their mucoadhesive properties resulted in increased brain uptake, with histopathological evaluation confirming their safety. 

Hadel *et al*. performed a study to investigate the occurrence of migraine, which is often accompanied by psychiatric disorders such as anxiety and depression (139). Eletriptan hydrobromide (EH) is not absorbed enough orally to reach the brain effectively. A nasal assemblesymptoms rapidly and effectively. The formulations were optimized using thin-film hydrolysis and factorial design to maximize zeta potential, entrapment efficiency, and particle size. According to in vitro studies, permeability was enhanced with a favorable residence time. Three months were required for EH emulsions to be stable. EH was effectively delivered through the nose to the CNS without causing any damage to the nasal mucosa in vivo, confirming their efficacy and safety.

In 2017, a study investigated rizatriptan benzoate’s anti-migraine potential by using SLNs to target brain regions (140). Critical formulation variables that affected RB-SLN fabrication were identified in the Plackett-Burman formulation. SLNs with a Box-Behnken design have been found to have a size of 220.4 2.3 nm, an entrapment efficiency of 71.8 1.9%, as well as a cumulative release rate of 45.9 2.7% after 8 hr. By determining the structure of the SLN using TEM images, XRD, thermal analysis, and FTIR spectroscopy, the SLN structure has been confirmed. Following oral administration for 2 hr, in vivo studies have demonstrated an 18.43-fold increase in brain uptake compared to free drugs. It was shown in pharmacodynamic studies of Swiss albino mice that RB-SLNs possess enhanced anti-migraine efficacy, suggesting their potential as migraine remedies. 

A 2023 study investigated dissolving microneedles containing caffeine and ergotamine, which were developed as a synergistic migraine treatment (141). Microneedles were created by incorporating drug-loaded Poly Lactic-co-Glycolic Acid (PLGA) nanospheres into a polymer matrix and integrating them into the polymer matrix. As a result of their narrow size distributions and good stability, the nanospheres were highly entrappable and controlled when released. Microneedles produced sustained release both *ex vivo* and *in vitro*, with antihyperalgesic activity and nontoxicity confirmed by serotonin and histopathology tests. A new 3D applicator effectively delivers intranasal migraine medications into the nasal cavity, making it a promising approach for treating the condition. Table 4 shows a summary of the other nanoformulations used for migraine. 

**Challenges and future directions in nanoformulation for migraine treatment**

Several challenges must be overcome to enhance migraine treatment through nanoformulation. Furthermore, research efforts are underway to identify innovative strategies for overcoming these challenges and advancing migraine therapy with nanoformulation (142). 

***Challenges***

Migraine therapy faces several challenges, one of which is ensuring that the drugs delivered to the CNS, which is the primary site of migraine pathology, are effective (143). The BBB limits therapeutic delivery to the brain parenchyma, which limits migraine medications’ effectiveness. Developing nanoformulations that can penetrate the BBB and deliver drugs to the desired target sites within the CNS is a challenging endeavor that requires innovative strategies to maximize penetration within the BBB while minimizing adverse effects off-target. A significant aspect of clinical translation is ensuring the safety and biocompatibility of nanoformulations (144). However, despite their advantages in drug delivery and targeting, there needs to be more concern regarding the long-term effects of NPs’ potential toxicity and immunogenicity on biological systems. To minimize the danger of adverse effects and guarantee patient safety, researchers must carefully evaluate the biocompatibility of nanocarriers and their degradation products. Increasing the cost-effectiveness, reproducibility, and quality control of nanoformulations for clinical use are significant challenges. Standardized protocols are crucial in transitioning from laboratory to large-scale production to ensure consistently high product quality and performance. Furthermore, a nanoformulation formulation method and manufacturing technique can impact its properties and performance, which require optimization and validation throughout development (47). Achieving regulatory approval for nanoformulations in light of their novelty and complexity presents a unique challenge. For regulators to evaluate nanoformulations for quality, safety, and efficacy and their comparability to conventional therapies, comprehensive preclinical and clinical data is required (145). Successful regulatory approval and clinical translation require demonstrating bioequivalence between nanoformulations and existing therapies, addressing potential immunogenicity concerns, and addressing biodistribution issues. As nanoformulations are developed and manufactured at high cost, widespread adoption and accessibility may be hindered, especially in places with limited resources. Regulatory compliance, nanomaterials, and specialized equipment can increase production costs, limiting patient access to innovative therapies (146). Consequently, maximizing manufacturing processes, reducing production costs, and streamlining regulatory procedures are crucial to ensuring migraine nanoformulations are affordable and accessible. 

***Future directions***

In the future, researchers should investigate novel strategies that will improve drug delivery to the central nervous system and enhance the penetration of the BBB. Multifunctional nanocarriers capable of actively reaching and crossing the BBB may be designed, as well as non-invasive treatment methods such as intranasal administration or focused ultrasounds that can open the BBB (13). Furthermore, advances in nanotechnology, including the development of stimuli-responsive and self-assembling NPs, could provide an innovative way to enhance BBB penetration within a patient’s brain and improve drug distribution within the body. Advancements in nanotechnology make it possible to target therapeutics precisely to the cells or molecular targets involved in migraine pathogenesis (147). In the future, nanoformulations may include surface modifications or targeting ligands to improve their affinity for diseased cells or receptors, improving therapeutic efficacy while minimizing off-target effects. Using receptor-mediated endocytosis and molecular recognition, researchers can develop nanocarriers to selectively deliver drugs to migraine-specific targets, such as inflammation mediators, trigeminal neurons, and glial cells (118). Researchers are pursuing the development of personalized nanoformulations designed based on the characteristics of patients and their responses to treatment. The system integrates patient-specific data, such as imaging findings, biomarker expression patterns, and genetic profiles, to design nanoformulations tailored to patients’ needs and disease characteristics (46). With the use of custom medicine approaches, migraine patients may receive more precise dosing regimens, suffer fewer adverse effects, and have better treatment outcomes, ultimately leading to a paradigm shift towards migraine precision therapy (148). When multiple therapeutic agents are combined in a single nanoformulation, beneficial synergistic effects can be achieved, and the therapeutic outcome can be improved. A combination of analgesics, anti-inflammatory agents, neuroprotectant agents, and neuromodulators may be incorporated into future migraine nanoformulations. A combination of drugs with complementary mechanisms enhances pain relief, reduces inflammation, and prevents migraine attacks more effectively than monotherapies (13). In addition, taking advantage of the co-delivery of therapeutic agents within nanoformulations and imaging probes may help monitor disease progression and treatment response in real-time, facilitating customized therapeutic interventions and improving patient care (109). Furthermore, nanotechnology provides opportunities to develop advanced diagnostic tools to diagnose, monitor, and prognosis migraine. Biomarkers, imaging agents, and biosensors based on NPs can give insight into the pathophysiology of disease, identify disease biomarkers, and provide non-invasive treatment response monitoring (146). A nanotechnology-enabled diagnostic that integrates specificity, sensitivity, and multiplexing capabilities may improve therapeutic decision-making, patient stratification, and migraine management. 

**Table 1:** Summary of the studies about curcumin nanoformulations used for migraine

**Nano formulation **	**Methods**	**Study model / Administration**	**Results**	**Ref.**
**Nano-curcumin and n-3 fatty acids**	Measurement of migraine attack frequency and serum levels of IL-1β; comparison between individual treatments and combination	Human	Significant reduction in migraine attack frequency and serum levels of IL-1β with combination vs individual treatments (*P*<0.001). After modifications, a significant reduction in IL-1β gene expression was observed between combination and individual groups (*P*<0.05)	(113)
**Liposomal curcumin and naproxen**	Evaluation of anti-oxidant and anti-nociceptive actions; comparison with naproxen alone or curcumin solution in rat migraine models	Rat / intraperitoneal and intravenous	Significant reduction in oxidative stress markers and pain with a combination of naproxen (2.8 mg/kg) and liposomal curcumin (2 mg/0.1 kg) vs naproxen alone (*P*<0.001)	(114)
**Omega-3 fatty acids and nano-curcumin**	Analysis of iNOS/COX-2 levels and clinical symptoms in 74 migraine patients	Human	Significant reduction in iNOS/COX-2 levels and clinical symptoms with omega-3 and nano-curcumin vs control (*P*<0.05). It is promising for migraine prevention	(115)

**Table 2:** Summary of the studies about Zolmitriptan nanoformulations used for migraine

**Nano formulation **	**Methods**	**Study model / Administration**	**Results**	**Ref.**
**Mucoadhesive nanoemulsions**	Evaluation of ZMT mucoadhesive nanoemulsion viability, drug content, morphology, zeta potential, permeation, and pharmacokinetics in mice	Mice / intravenous and nasal	In mice, enhanced brain AUC and reduced Tmax were observed with mucoadhesive nanoemulsion, indicating improved brain delivery and rapid action compared to intravenous and nasal solutions	(112)
**Mucoadhesive polymeric NPs**	Assessment of ZMT NPs for intranasal administration in mice; evaluation of bioavailability, migraine symptom reduction	Mice / intranasal	Compared to oral administration, ZMT NPs significantly increased absolute bioavailability (193%) and improved nasal-to-brain transport. Reduced migraine symptoms were observed in mice, suggesting the efficacy of intranasal delivery for migraine treatment	(116)
**Chitosan NPs**	Pharmacokinetic study in Wistar albino rats receiving nasal sprays of chitosan NPs containing ZMT	Wistar albino rats / Nasal spray	Significant increase in plasma and brain tissue drug levels with chitosan NP nasal spray, indicating rapid absorption and potential efficacy in treating migraine	(117)
**Solid Lipid Nanoparticles (SLNs)**	Investigation of SLNs for ZMT delivery across the blood-brain barrier (BBB) in male Wistar rats	Male Wistar rats / intranasal	SLNs exhibited favorable characteristics and enhanced pharmacokinetic parameters, indicating improved bioavailability and permeation of ZMT. Promising intranasal delivery approach for bypassing hepatic metabolism and enhancing drug efficacy for migraine treatment	(118)
**PLGA/poloxamer NPs**	Development and characterization of ZMT-loaded NPs; evaluation of brain uptake in Swiss albino mice	Swiss albino mice / Intranasal	Optimized NPs showed increased brain uptake and enhanced anti-migraine potential compared to free drugs, indicating the potential efficacy of ZMT-loaded NPs in treating migraine	(106)
**Polymeric carriers**	Characterization and *in vivo* brain uptake evaluation of ZMT-loaded nanocarriers in rat migraine models	Rat / Intranasally	Improved brain uptake was observed with ZMT-loaded nanocarriers administered intranasally, suggesting their potential as migraine treatment systems	(119)
**Liposomes**	Development and characterization of ZMT/chitosan nanostructured liposomes; evaluation of pharmacological effectiveness	*Ex vivo*	ZMT/CT NLCs displayed good performance with high entrapment efficiency and stability, suggesting potential for improving drug efficacy in migraine treatment	(122)
**Liposomes**	Formulation and characterization of ZMT-loaded nanoliposomes; evaluation of mucoadhesive gel properties	*Ex vivo*	Nanoliposomes integrated into mucoadhesive gel exhibited promising characteristics, including good release, permeation, and non-toxic effects. This suggests the potential for effective nose-to-brain delivery of ZMT for migraine treatment	(125)

**Table 3:** Summary of the studies about Sumatriptan nanoformulations used for migraine

**Nano formulation **	**Methods**	**Study model / Administration**	**Results**	**Ref.**
**Micelles**	Description of protocol for preparing sumatriptan-loaded micelles for migraine treatment; characterization of micelles	Oral, transdermal, and parenteral administration	Micelles provide potential noninvasive delivery for migraine treatment, offering advantages of oral, transdermal, and parenteral administration. Hydrophobic regions of diblock polymer hold the drug, while hydrophilic regions provide conformational stability in aqueous environments, making micelles useful in various administration routes	(127)
**NLCs**	Development of NLCs for nose-to-brain delivery of sumatriptan; optimization of formulation parameters; *in vivo* pharmacokinetic evaluations in rats	Rat /Intranasal	Optimized NLCs demonstrated significant Drug-Targeting Efficiencies (DTEs) and Direct Transport Percentages (DTPs) in rats, indicating the potential for brain delivery of sumatriptan	(128)
**Chitosan-coated nanoliposomes**	Formulation optimization and pharmacokinetic evaluation of chitosan-coated nanoliposomes for enhanced bioavailability of sumatriptan succinate	White New Zealand male rabbits / Internasal	Chitosan-coated NLs enhanced sumatriptan absorption, leading to faster absorption and improved systemic delivery compared to soluble forms. Animal studies showed better kinetic behavior of liposomal and chitosan formulations, indicating their potential for improving sumatriptan bioavailability and efficacy in migraine treatment	(129)
**PBCA NPs and SS-loaded BSA-ApoE NPs**	Optimization of PBCA NPs and SS-loaded BSA-ApoE NPs for brain delivery of sumatriptan succinate; comparison of brain uptake and antimigraine potential in rats	Rats /Internasal	SS-loaded BSA-ApoE NPs showed higher brain uptake and superior antimigraine potential than PBCA NPs in rats, indicating the potential for improved brain targeting of sumatriptan succinate in migraine treatment	(131)
**Liposomal **	Investigation of combination treatment with liposomal curcumin and sumatriptan in experimental migraine model induced by nitroglycerin in rats	Rats /Intravenous	Liposomal curcumin combined with sumatriptan significantly reduced oxidative stress levels, suggesting potential as an optimal treatment method for migraine. The superior antioxidative properties of liposomal curcumin compared with curcumin solution indicate its promise in migraine therapy, warranting further research	(132)
**Nanoemulsion**	Development of stable Nano Emulsion (NE) containing copaiba oil and biopolymers; evaluation of NE properties and *in vivo* efficacy in zebrafish	Zebrafish / Intranasal	Stable NE prolonged sumatriptan release for over 24 hr and demonstrated safety and potential efficacy in zebrafish, suggesting its potential in treating migraine pain	(133)
**Magnetic porous carriers**	Synthesis of magnetic porous carriers based on tin oxide nanocrystals for efficient and controlled drug release of sumatriptan; characterization and stability testing	*Ex vivo*	Optimal drug efficiency (70%) was achieved with magnetic porous carriers based on tin oxide nanocrystals, demonstrating efficient and controlled drug release. *In vitro* studies and stability testing confirmed the suitability of carriers for *in vivo* applications, suggesting their potential for enhancing sumatriptan delivery in migraine treatment	(134)

**Table 4:** Other nanoformulations used for migraine

**Nano formulation used**	**Methods**	**Study model / Administration**	**Results**	**Ref.**
**Aerosol drug delivery system for flunarizine dihydrochloride**	Investigation of compatibility study and formulation suitability	*Ex vivo*	The Aerosol drug delivery system for flunarizine dihydrochloride is confirmed to be suitable with compatibility study, suggesting potential for improving solubility and therapeutic effect	(109)
**PEGylated lipid-polymer hybrid nanoparticles**	Production of ergotamine and caffeine hybrid NPs; characterization and evaluation of controlled release, entrapment efficiency, and permeability	Mice / Internasal	NPs exhibited favorable characteristics, including good stability, small size, controlled release profile, high entrapment efficiency, sustained release over 48 hr post-intranasal administration, and significant brain uptake. They confirmed the safety and anti-hyperalgesic effects, indicating the potential for treating migraine	(107)
**Solid lipid nanoparticles (SLNs) + mucoadhesive ** ***in situ*** ** gel**	Preparation and optimization of SLNs loaded with Almotriptan Malate (ALM); development of mucoadhesive *in situ* gel containing the optimized SLNs; evaluation of delivery system efficacy and safety	Rat/Internasal	SLN-loaded *in situ* gel rapidly delivered ALM into the brain, with promising results in both *in vitro *and* in vivo *studies and favorable toxicological risk profiles, indicating potential for future clinical trials and positive outcomes in human migraine treatment	(137)
**Chitosan-coated nanostructured lipid carriers (NLCs)**	Formulation optimization and evaluation of chitosan-coated NLCs for Almotriptan maleate (ALM) delivery through nasal mucosa	Albino rabbits/Internasal	Formulated chitosan-coated NLCs showed favorable properties and increased brain uptake, with histopathological evaluation confirming safety, suggesting potential for effective delivery of ALM through nasal mucosa for migraine treatment	(138)
**Solid lipid nanoparticles (SLNs) for rizatriptan benzoate**	Identification of critical formulation variables and optimization of SLNs for rizatriptan benzoate delivery; characterization and evaluation of brain uptake and anti-migraine efficacy	Swiss albino mice/Internasal	Optimized SLNs demonstrated favorable characteristics and enhanced brain uptake, with significant anti-migraine efficacy demonstrated in pharmacodynamic studies, indicating their potential as migraine remedies	(140)
**Dissolving microneedles containing caffeine and ergotamine**	Development of dissolving microneedles containing caffeine and ergotamine for migraine treatment; characterization and evaluation of sustained release and safety	*In vitro* and *ex vivo*	Microneedles produced sustained release, antihyperalgesic activity, and confirmed safety. A new 3D applicator effectively delivered intranasal migraine medications into the nasal cavity, suggesting a promising approach for treating migraine	(141)

## Conclusion

Nanoformulation emerges as a promising frontier in addressing the complex and debilitating nature of migraine, offering a potential solution to overcome the limitations of conventional treatments. By encapsulating therapeutic agents within nanocarriers and modulating their properties, nanoformulation optimizes drug delivery, enhances bioavailability, and minimizes adverse effects, providing superior efficacy compared to traditional medications, lifestyle changes, and behavioral therapies. While conventional treatments may offer symptomatic relief, nanoformulation stands out for its ability to target specific mechanisms underlying migraine attacks, such as neuroinflammation or neurotransmitter imbalances, and enable personalized medicine approaches tailored to individual patient needs. Despite challenges related to safety, biocompatibility, scalability, and regulatory approval, ongoing research efforts continue to advance the field of nanoformulation for migraine therapy. Future directions include enhancing blood-brain barrier penetration, developing nanotechnology-enabled diagnostics, and incorporating personalized medicine approaches to optimize patient outcomes. In conclusion, nanoformulation holds the potential to revolutionize migraine treatment, offering patients personalized and precise therapies that improve their quality of life and overall well-being.

## Data Availability

The datasets generated and/or analyzed during the current study are available from the corresponding author upon reasonable request.

## Declaration

We have not used any AI tools or technologies to prepare this manuscript.
